# Efficacy and Safety of Mesenchymal Stem Cells in Treatment of Complex Perianal Fistulas: A Meta-Analysis

**DOI:** 10.1155/2020/8816737

**Published:** 2020-11-21

**Authors:** Fang Cheng, Zhong Huang, Zhi Li

**Affiliations:** Division of Gastroenterology, Zigong First People's Hospital, Sichuan, China

## Abstract

Complex perianal fistula is a highly debilitating and difficult to treat condition. Local mesenchymal stem cell (MSC) therapy for perianal fistula has shown considerable promise but still remains controversial. Therefore, we performed the meta-analysis to evaluate the efficacy and safety of local MSC therapy for complex perianal fistula. PubMed and Embase databases were searched for published randomized clinical trials (RCTs) that reported local MSC therapy for complex perianal fistulas. The effectiveness and safety data analysis was conducted using RevMan5.3. Subgroup analyses were performed based on the characteristics of the studies. Seven RCTs with 730 participants were included. Local MSC treatment showed significantly higher healing rate (HR) of perianal fistulas compared to control (odds ratio (OR) = 2.03; 95% confidence interval (CI) 1.50, 2.74; *P* < 0.00001). MSCs combined with fibrin glue therapy can improve the HR compared with fibrin glue alone (OR = 3.27; 95% CI 1.15, 9.28; *P* = 0.03). Subgroup analyses showed that local therapy can improve the HR in patients with perianal fistulas associated with Crohn's disease (CD) (OR = 2.05; 95% CI 1.41, 3.00; *P* = 0.0002) and cryptoglandular origin (no-Crohn) (OR = 2.98; 95% CI 0.86, 10.29; *P* = 0.08). The pooled OR for studies that combined reepithelialization of the external opening with pelvic magnetic resonance imaging (MRI) to evaluate the healing of fistulas was 1.77 (95% CI 1.28, 2.45; *P* = 0.0006). The pooled OR for studies where fistula healing was defined as complete reepithelialization of external openings was 5.92 (95% CI 1.34, 26.15; *P* = 0.02). Both autologous MSCs (OR = 3.19; 95% CI 1.05, 9.65; *P* = 0.04) and allogeneic MSCs (OR = 1.97; 95% CI 1.34, 2.91; *P* = 0.0006) can obtain higher HR for perianal fistula compared with control. The adipose-derived MSC group can obtain higher HR than the control group (OR = 2.29; 95% CI 1.38, 3.79; *P* = 0.001). There were no significant differences in adverse events (AEs) (OR = 1.06; 95% CI 0.71, 1.59; *P* = 0.77). None of the adverse events was judged to be related to MSCs. Our study supported that local MSC therapy alone or combined with fibrin glue is safe and efficacious for complex perianal fistula. In the future, more RCTs are needed to confirm this conclusion.

## 1. Introduction

Perianal fistula has an incidence of 1.1 to 2.2 per 10 000 persons per year, which continues to increase in incidence for unknown reasons and results in a significant burden to the healthcare system [1]. It is also among the most common illnesses affecting people and negatively impacts the quality life of patients. Gastroenterology association classified fistula into simple and complex fistula. Complex fistulas are extrasphincteric, suprasphincteric, high intersphincteric, or high transsphincteric or have multiple external openings or are associated with an abscess or with rectovaginal fistula or with anorectal stenosis. Complex perianal fistula is the worst scenario; it presents a difficult management and remains a great challenge for clinicians. Patients with complex perianal fistulas often suffer from fecal incontinence and high recurrence rates. Particularly in patients with fistula associated with Crohn's disease (CD), high recurrence and the vast complication induced by fistulas, such as secondary infection, abscess formation, and organ system function impaired, significantly reduce quality of life [2]. Some studies showed that the risk of carcinogenesis among patients with Crohn's fistulas has increased both in intraintestinal tumors and extraintestinal tumors [3–5]. Besides that, perianal fistula has also been associated with the development of anal cancer and can affect about 28% of patients in 20 years after diagnosis [6]. The exact pathogenesis of complex perianal fistula is unknown. It is an immune-mediated chronic recurrent disease, and it is a pathological condition that has different options of treatment. The treatment goal of perianal fistula is to stop fistula from draining, relieve symptoms of patients, avoid complications or the fecal incontinence, and achieve a durable closure finally [7, 8]. The existing clinical managements for complex perianal fistula (either of cryptoglandular origin or associated with CD) include conventional medications (e.g., antibiotics and immunomodulators) and antitumour necrosis factor agents (anti-TNF) (e.g., infliximab, adalimumab, and certolizumab), surgical treatments, or fecal diversion. A phase III clinical trial showed a superiority of vedolizumab over placebo for induction and maintenance of remission on luminal CD [9]. Some studies also support that ustekinumab may improve the HR of perianal fistulas [10, 11]. Unfortunately, existing treatments with high rate of recurrence and are difficult to maintain sustained fistula closure.

Cycle through numerous immunomodulators leads to significant side effects. Biological treatment may increase the risk of opportunistic infection. Limited surgical procedures often result in fecal incontinence. Combined surgical and medical treatment is recommended by clinical guidelines [12], and this therapy strategy is better than either treatment alone in achieving fistula healing. But the highest HR is limited about 50% and with a high relapse rate. Fecal diversion may be used to manage perianal fistulas, recurrence is high, and most patients will suffer from rectal resection eventually. So, there are clear limitations with current medical and surgical options. This depressing condition has aroused great interest in finding better treatment. Anti-inflammation and immunomodulation are severe challenges for the closure and fibrosis of fistulas for clinical physicians. Novel therapeutic approaches without a risk of incontinence were explored urgently.

In recent years, some studies have demonstrated that local MSC treatments have shown notable promising results in the treatment of perianal fistulas [13–27]. MSCs are one of the most popular multipotent stem cells. The lack of substantial immunogenicity of MSCs allowed use across human leukocyte antigen (HLA) barriers. Some studies have showed that MSCs function not to induce immune rejection after transplantation [28]. They can be isolated from different tissues such as bone marrow, adipose tissue, and umbilical cord and are expanded under in vitro conditions to obtain in large quantities. MSCs have high proliferation and differentiation capacity and are characterized by multilineage differentiation, anti-inflammation, and immunomodulation. They can repair damaged tissues to promote tissue healing and may achieve long-term healing of fistula, significantly improving the quality of life of patients. Taken together, it is considered a promising therapeutic option for treating complex perianal fistulas. So, we aim to carry out a meta-analysis of RCTs to further evaluate the effectiveness and safety of MSCs in the treatment of complex perianal fistulas.

## 2. Materials and Methods

This review was prepared according to the Preferred Reporting Items for Systematic Reviews and Meta-Analysis (PRISMA) guideline [29].

### 2.1. Search Strategy

A comprehensive search of PubMed and Embase were conducted through March 2020, with no restriction. The search strategy included the following medical keywords: inflammatory bowel disease, Crohn's disease, Crohn disease, mesenchymal stem/stromal cells, stem cell, stromal cell, perianal fistula, Crohn's perianal fistula, and cryptoglandular perianal fistula. Two reviewers (FC and ZH) independently assessed the titles and abstracts of the studies that met the eligibility criteria for inclusion.

### 2.2. Study Selection

Inclusion criteria were (1) type of study: human subjects; (2) RCTs; (3) object of the study: patients with complex perianal fistulas (either of cryptoglandular origin or associated with CD); (4) interventions: local therapy with MSCs alone or MSCs plus intralesional fibrin glue for complex perianal fistula; and (5) outcome: efficacy and/or safety was reported. Exclusion criteria were (1) nonhuman studies; (2) reviews, case reports/series, one-arm clinical trials, review articles, conference abstracts, letters to the editor, or studies for which the full text was unavailable; and (3) efficacy of MSCs as systemic infusion for perianal fistulas. Based on the criteria, two investigators (FC, ZH) scrutinized titles and abstracts to exclude irrelevant studies and reviewed the full text to further exclude unrelated studies.

### 2.3. Data Extraction and Quality Assessment

Data extraction was independently conducted by two investigators (FC, ZH). The following information was collected from eligible studies: (1) first author; (2) publication date and location; (3) patient demographics (number of patients, age); (4) type of perianal fistula; (5) the number of each group; (6) type and source of MSCs; (7) outcome assessment; (8) dosage and modalities of administration; (9) follow-up time and recurrence; (10) refractory disease; and (11) adverse events (AEs). Basic demographics and clinical characteristics of each included study are summarized in Table 1. Cochrane risk of bias tool was used to evaluate the bias risk of each enrolled study by two investigators [30]. The following were assessed for each study: selection bias, performance bias, detection bias, attrition bias, reporting bias, and other bias. Each item was classified as low risk, high risk, or unclear. The risk of bias was determined by taking all items together in a risk bias graph. Any disagreement was resolved through discussion.

### 2.4. Statistical Analysis

The healing rate (HR) of complex perianal fistula was regarded as the main endpoint. We analyze and manage all data analyses through Review Manager 5.3. Odds ratio (OR) and related 95% CIs were calculated to compare treatment with control groups. Statistical heterogeneity between the clinical trials was assessed via *I*^2^ statistics (*I*^2^ ≥ 50%, <50% indicating substantial and low heterogeneity, respectively). For *I*^2^ < 50%, a fixed-effects model was applied. Otherwise, we used a random-effects statistical model. To identify heterogeneity, a sensitivity analysis with omission of one study at a time was performed. A value of *P* < 0.05 was considered statistically significant.

## 3. Results

### 3.1. Literature Search and Quality Assessment

**Figure 1:**
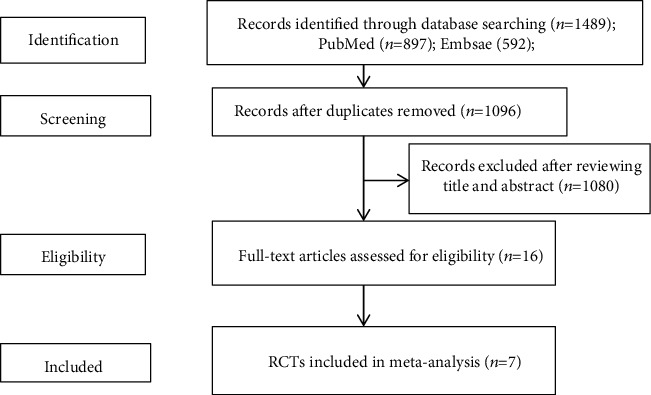
Flow diagram of included and excluded studies in this meta-analysis.

**Figure 2:**
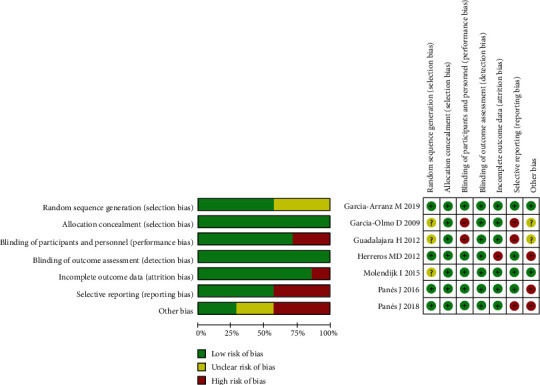
Risk of bias of the RCTs included in the meta-analysis.

A total of 1489 references were obtained in an electronic database. After removing duplicates, we obtained a total of 1096 records. After carefully reviewing the full-text studies, 1080 studies were further excluded. The remaining 16 records were assessed for eligibility through scrutinizing the full text. Finally, seven RCTs were included in our meta-analysis [14, 16, 17, 22, 23, 26, 27]. The detailed literature screening process is shown in Figure 1. Four studies were performed in Spain [14, 16, 17, 27]. One study was performed in Netherlands [22]. Two studies were performed in Israel [23, 26]. Six studies used adipose-derived MSCs [14, 16, 17, 23, 26, 27]. One study used bone-marrow-derived MSCs [22]. Four studies used autologous MSCs [14, 16, 17, 27]. Three studies used allogeneic MSCs [22, 23, 26]. Fistula healing was defined as complete reepithelialization of external in two studies [14, 27] and was defined as reepithelialization of the external opening and absence of collection > 2 cm by MRI in five studies [16, 17, 22, 23, 26] Four studies compared MSCs plus fibrin glue with fibrin glue alone [14, 16, 17, 27]. Two studies included patients with complex perianal fistulas associated with CD and cryptoglandular origin [14, 16]. Three studies included patients with complex perianal fistulas associated with CD [22, 23, 26]. Two studies included patients with complex perianal fistulas that were of cryptoglandular origin [17, 27]. Six studies included complex perianal fistulas that were refractory [14, 16, 17, 22, 23, 26]. The methodological quality of the included studies was regarded as acceptable, as most of the domains of the included studies were ranked as low. Low risk of bias was mostly detected in selection bias, performance bias, detection bias, attrition bias, and reporting bias. High risk of bias mostly occurred in reporting bias and other bias. Unclear risk of bias mostly occurred in selection bias and other bias. A summary of the risk of biases of the included studies is presented in Figure 2.

### 3.2. Efficacy of MSCs for Complex Perianal Fistulas

**Figure 3:**
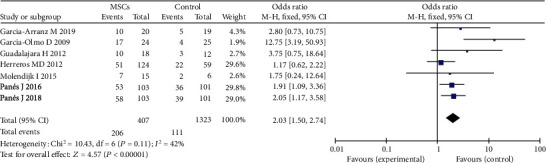
Efficacy of the MSC/MSC+fibrin glue group vs. the control group (fibrin glue or saline solution) in complex perianal fistulas.

The healing of fistula was the primary endpoint of our meta-analysis. Seven RCTs were included. There were 407 participants in the MSC group and 323 participants in the control group. In a fixed-effects model, compared with the control group (intralesional injection of fibrin glue or saline solution), the local therapy with MSCs or MSC plus fibrin glue group obtained higher HR of perianal fistula (50.6% versus 34.4%; OR = 2.03; 95% CI 1.50, 2.74; *P* < 0.00001) (Figure 3).

### 3.3. Efficacy of MSCs for Complex Perianal Fistulas (MSCs+Fibrin Glue vs. Fibrin Glue Alone)

**Figure 4:**
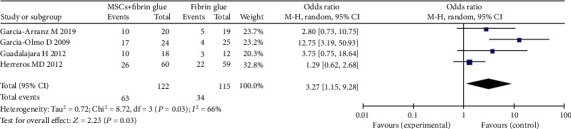
Efficacy of MSCs plus fibrin glue vs. fibrin glue alone treatment in complex perianal fistulas.

The fibrin glue was being studied as a vehicle for the MSCs in regenerative medicine, and it is capable of stimulating the cellular adhesion and growth. Combined stem cells with fibrin glue treatment for perianal fistula, angiogenic action of the fibrin matrix [31], and the differentiation ability of MSCs can promote the healing of fistula [32]. However, there is no sufficient evidence for recommendation of intralesional fibrin glue plus MSCs for fistula. In our study, we analyzed the efficacy of MSCs plus fibrin glue for perianal fistulas. Four studies were recruited [14, 16, 17, 27], with high heterogeneity between the studies (*I*^2^ = 66%). There were 122 participants in the MSC plus fibrin glue group and 115 participants in the fibrin glue group. In a random-effects model, MSCs plus fibrin glue were more effective for fistula healing than fibrin glue alone (51.6% versus 29.6%; OR = 3.27; 95% CI 1.15, 9.28; *P* = 0.03) (Figure 4). Sensitivity analysis showed that one study [14] had a major impact on this heterogeneity. After excluding the study, the heterogeneity decreased significantly. The analysis results also indicated that the MSC plus fibrin glue group had higher levels of HR compared with the fibrin glue group.

### 3.4. Efficacy of MSCs for Different Types of Complex Perianal Fistula (CD or Non-CD)

**Figure 5:**
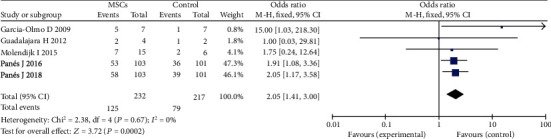
Efficacy of MSCs for perianal CD.

**Figure 6:**
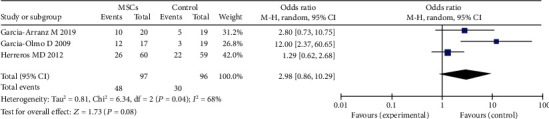
Efficacy of MSCs for complex perianal fistulas of no-Crohn.

Five studies [14, 16, 22, 23, 26] were recruited into this meta-analysis to detect the efficacy of MSC therapy for perianal CD. There were 232 participants in the MSC group and 217 participants in the control group (fibrin glue or saline solution). In a fixed-effects model, the result showed that the MSC group was more effective for fistula healing than control (53.9% versus 36.4%; OR = 2.05; 95% CI 1.41, 3.00; *P* = 0.0002) (Figure 5); three studies [14, 17, 27] were recruited into this meta-analysis to detect the efficacy of MSC therapy for complex perianal of no-Crohn. There were 97 participants in the MSC plus fibrin glue group and 96 participants in the fibrin glue group. Our results indicated that local therapy with MSCs was associated with improved HR as compared with the control group (49.5% versus 31.2%; OR = 2.98; 95% CI 0.86, 10.29; *P* = 0.08). (Figure 6).

### 3.5. Efficacy of MSCs for Complex Perianal Fistulas (Different Evaluation Methods for the Healing of Fistula)

**Figure 7:**
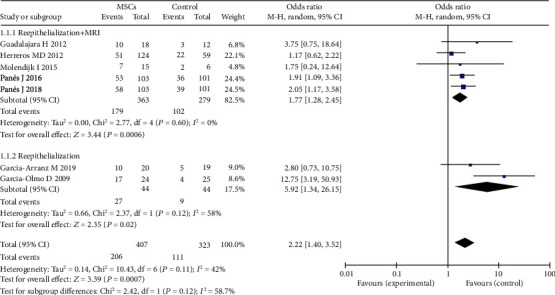
Different evaluation methods for the healing of fistulas.

In our meta-analysis, some studies [16, 17, 22, 23, 26] defined fistula healing as reepithelialization of the external opening and absence of collection > 2 cm by magnetic resonance imaging (MRI). Some studies [14, 27] defined fistula healing as complete reepithelialization of external opening. Pelvic MRI is a noninvasive, highly accurate examination for the diagnosis and classification of fistula. Therefore, MRI is considered to be the imaging gold standard for fistulas [33]. Using MRI to evaluate the fistula healing in five trials, including 363 patients treated with MSCs, the pooled analysis showed that the MSC group obtained higher HR than the control group (fibrin glue or saline solution) (49.3% versus 36.6%; OR = 1.77; 95% CI 1.28, 2.45; *P* = 0.0006). Fistula healing was defined as complete reepithelialization of external openings in two trials; the pooled analysis also showed that the MSC plus fibrin glue group obtained higher HR than the fibrin glue group (61.4% versus 20.5%; OR = 5.92; 95% CI 1.34, 26.15; *P* = 0.02) (Figure 7). With high heterogeneity between the studies, healing was defined as reepithelialization. Sensitivity analysis showed that one study [14] had a major impact on this heterogeneity. After excluding that study, the heterogeneity decreased significantly.

### 3.6. Different Sources of MSCs for the Treatment of Complex Perianal Fistulas

**Figure 8:**
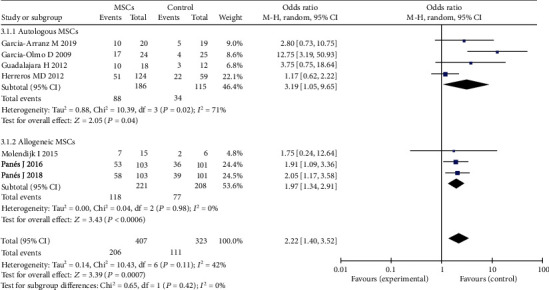
Different sources of MSCs for the treatment of complex perianal fistulas.

Four of the seven studies reported autologous-derived stem cells for the treatment of complex perianal fistula [14, 16, 17, 27]; there were 186 participants in the autologous MSC plus fibrin glue group and 115 participants in the fibrin glue group. Three of the seven studies reported allogeneic-derived stem cells for complex perianal fistula [22, 23, 26]; there were 221 participants in the allogeneic MSC group and 208 participants in the saline solution group. The random-effects model was applied. Regarding the effects of autologous MSC therapy, the MSC experimental group had a high HR compared to the control group (fibrin glue group) (47.3% versus 29.6%; OR = 3.19; 95% CI 1.05, 9.65; *P* = 0.04); regarding the effects of allogeneic MSC therapy, the allogeneic adipose-derived stem cell group also had a high HR compared to the control group (saline solution group) (53.4% versus 37.0%; OR = 1.97; 95% CI 1.34, 2.91; *P* = 0.0006) (Figure 8). Regarding the efficacy of autologous MSC therapy, there was high heterogeneity between the studies. Sensitivity analysis showed that one study [14] had a major impact on this heterogeneity. After excluding that study, the heterogeneity decreased significantly.

### 3.7. Different Cell Types of MSCs for the Treatment of Complex Perianal Fistulas

**Figure 9:**
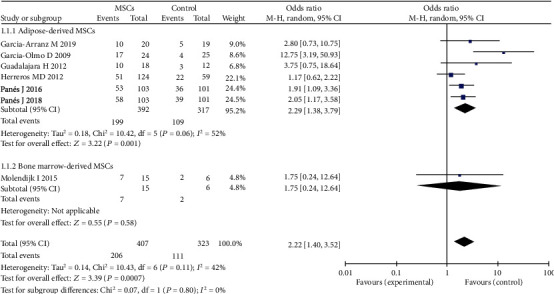
Different cell types of MSCs for the treatment of complex perianal fistulas.

MSCs can be obtained from the adipose tissue or bone marrow. Six of the seven studies reported adipose-derived MSCs [14, 16, 17, 23, 26, 27]; there were 392 participants in the adipose-derived MSC group and 317 participants in the control group (fibrin glue or saline solution). One study reported bone marrow-derived MSCs [22]. The pooled analysis showed that the adipose-derived MSC group (50.8% versus 34.4%; OR = 2.29; 95% CI 1.38, 3.79; *P* = 0.001) and bone marrow-derived MSC group (46.7% versus 33.3%; OR = 1.75; 95% CI 0.24, 12.64; *P* = 0.58) can obtain higher HR than the control group (Figure 9). However, only one study was included in the bone marrow-derived MSC therapy group. Regarding the efficacy of adipose-derived MSC therapy, there was high heterogeneity between the studies. Sensitivity analysis showed that one study [14] had a major impact on this heterogeneity. After excluding that study, the heterogeneity decreased significantly.

### 3.8. Adverse Events

**Figure 10:**
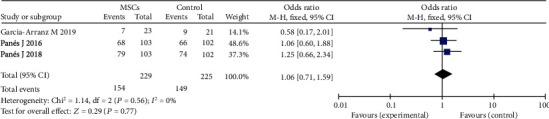
Pooled OR of adverse events.

This study included three individual articles that performed RCTs to determine the safety of perianal fistulas [23, 26, 27]; there were 229 participants in the MSC group and 225 participants in the control group. There was no difference in AEs between the MSC and control group (OR = 1.06; 95% CI 0.71, 1.59; *P* = 0.77 (Figure 10). Our study showed that there was no increase in adverse or serious adverse events with MSCs as compared with control. No MSC-related adverse events (AEs) have been reported so far. Common AEs such as anal pain, anal bleeding, fever, abdominal pain, diarrhea, and perianal abscess are mostly associated with local injection procedures of MSCs rather than MSCs themselves.

## 4. Discussion

In recent years, there is a rapidly growing interest in stem cell therapy for patients with perianal fistulas. Stem cells are a unique group of undifferentiated cells that have capacity of self-renewal, and they can be broadly categorized as embryonic or adult-derived stem cells. Among adult stem cells, the best defined cells are the hematopoietic stem cells (HSCs), MSCs, and intestinal stem cells. MSCs are nonhematopoietic multipotent stem cells that have the capacity to differentiate into a limited array of differentiated cell types of mesodermal lineage including chondrocytes, osteoblasts, and adipocytes under different microenvironmental conditions, culture media, and supplements. Their properties include in vitro proliferation with plastic-adherent properties bearing fibroblast-like morphology, the expression of MSC markers and stem cell–specific genes, and the ability to form colonies and differentiate into various cell lineages. Given that MSCs are able to downregulate immune responses and anti-inflammatory properties and promote tissue healing, they are most commonly used to treat perianal fistulas. Multiple studies have evaluated MSCs to determine the safety and efficacy of treating perianal fistula. The first report that explored the use of MSCs as a treatment for perianal fistula was a case report in 2003 [34]; a perianal CD was successfully healed after local MSC therapy. There were more and more phase I/II studies that appeared. In 2016, there was a large sample RCT study involving 212 CD patients, conducted in 49 hospitals in 7 European countries and Israel. A greater proportion of patients treated with Cx601 vs. placebo achieved remission at 24 weeks. The benefit over placebo was sustained 52 weeks after local injection [23]. There was a RCT that indicated that after one year of follow-up, no differences were found between patients receiving cells and those of the control group [27]. These studies are encouraging, but the results are still controversial. The explanation for this controversial result may be due to the different sources, different differentiation properties and regeneration capacity of MSCs, and different cell isolation and culture and the patient's age, severity of the disease, and other factors may also affect the efficacy of MSCs. To the best of our knowledge, this was the first meta-analysis of RCTs on local MSC therapy for complex perianal fistulas (either of cryptoglandular origin or associated with CD).

Our meta-analysis demonstrated that MSC alone or combined with fibrin glue treatment for complex perianal fistula (CD or non-CD) is effective and safe in seven RCTs. Compared with the control group, local therapy with MSCs or the MSC plus fibrin glue group obtained higher HR of perianal fistulas (OR = 2.03; 95% CI 1.50, 2.74; *P* < 0.00001) and can favor long-term and sustained fistula healing. These long-term improvements can be explained by the biological action of MSCs such as multilineage differentiation, anti-inflammation, and immunomodulation [35, 36]. Despite that, the precise mechanisms of perianal fistula are not known. Most studies indicated both innate immunity and adaptive immunity play a part in disease pathogenesis. MSCs can achieve the reconstruction of intestinal immunity, to avoid immune-mediated intestinal inflammation and may achieve long-term healing of perianal fistulas, significantly improving the quality of life of patients. Fibrin glue is not considered a cytotoxic product and is capable of stimulating the cellular adhesion and growth, being studied as a vehicle for the MSCs in regenerative medicine. There were only few studies that reported that the HR was higher when MSCs were combined with fibrin glue [37]. Whether there was any promotion effect of the MSC plus fibrin glue therapy remains unknown. In our study, compared with the fibrin glue alone group, the fistula HR of patients in the MSC plus fibrin glue group increased thrice (OR = 3.27; 95% CI 1.15, 9.28; *P* = 0.03). And there also was a study about autologous MSCs, applied in a bioabsorbable matrix of fibrin glue for the treatment of perianal fistulas in patients with successfully healed CD. We should that the think angiogenic action of fibrin glue and the differentiation ability of MSCs may have a synergistic effect on the healing of fistulas. Therefore, in the local treatment of MSCs, fibrin glue can be used as a mediator and promote the healing of fistulas. In our meta-analysis, MSC therapy was more effective for perianal CD healing than control (53.9% versus 36.4%; *P* = 0.0002), with a statistically significant difference (*P* < 0.05). The pooled OR for studies about MSC therapy for non-CD perianal fistulas was 2.98 (49.5% versus 31.2%; *P* = 0.08), with no statistically significant differences (P>0.05). Up to now, this was the first meta-analysis of RCTs on local MSC therapy for complex perianal fistulas (either of non-CD or associated with CD). In our study, MSCs have shown more promising results in fistulas associated with CD. The explanation for this result is that in our included studies, MSCs were used alongside anti-TNF in most CD patients and they share common targets and may have a synergistic effect on the healing of fistulas. In the future, well-designed RCTs will be needed to confirm this conclusion and comparison of the efficiency of MSC transplantation in the case of different types of perianal fistula. Standardization is crucial to assess the efficacy of current and future local MSC treatment strategies. MRI is the imaging gold standard for fistulas, and it can better reflect the inflammation inside the fistulas, and it also facilitates early detection of fistula recurrence. In our study, when MRI was used to assess the healing of the treated fistula, the HR was decreased. But it was still higher than that of the control group (OR = 1.77; 95% CI 1.28, 2.45; *P* = 0.0006). This result reminded us that the HR may be overestimated when the fistula healing was only defined as complete reepithelialization of external opening. Therefore, with the goal of optimizing this emerging therapy, we need more objective definitions of the healing of perianal fistulas (e.g., endoscopy and MRI) in the future studies.

In our study, autologous and allogeneic MSCs can improve the HR compared with control. The pooled OR for studies about autologous MSC therapy was 3.19 (95% CI 1.05, 9.65; P = 0.04) and about allogeneic MSCs was OR = 1.97 (95% CI 1.34, 2.91; *P* = 0.0006). The cell samples are derived from the patient's own body, safety problems were not anticipated, and the chance of stimulating an immunological response is practically none. Another point to consider is that the survival of autologous MSC in the body is superior when compared to a material from a donor. The risk of allogeneic-derived MSC being rejected by immunocompetent patients is greater [38]. So, autologous-derived MSCs are the optimum donor for treatment of perianal fistulas. But we should know autologous MSCs are not immediately available upon request because isolation and expansion of MSCs to sufficient numbers of cells require weeks, resulting in treatment delay. And disease-related effects on autologous MSCs must be taken into account. In the future, we need more studies to consider the timeliness and cost-effectiveness of treatment to optimize the treatment. MSCs can be isolated from various tissues including the bone marrow, adipose tissue, and human umbilical cord. Due to the easy gain property of adipose tissue-derived stem cells (ADMSCs) and the low immunogenicity property of bone marrow-derived stem cells (BMSCs) compared with other stem cells, both ASCs and BMSCs are the main stem cells to treat fistulas. Our study showed that both adipose-derived MSCs and bone marrow-derived MSCs can improve the HR. But only one bone marrow RCT was included in our study. This is the limitation of our study. Some studies showed that ADMSCs are superior to BMSCs in terms of immunosuppressive capacity. The subcutaneous adipose tissue is considered an easily accessible source of large amounts by minimally invasive procedures (aspiration or liposuction). Liposuction is considered low-invasive and inexpensive and provides an adequate number of cells even in small amounts [39]. Obtaining adipose tissue minimizes side effects on donors (regardless whether a patient or healthy donor). To obtain bone-marrow MSCs in some special donors such as patients with a history of myocardial infarction is dangerous. These facts suggest that there should be more attempts to make use of adipose tissue as a source of MSCs. Limitations of current studies and the optimal dosage of MSCs for the treatment of fistula remained unclear. Molendijk et al. [12] reported that administration of 3 × 10^7^ MSCs resulted in higher fistula healing compared with 9 × 10^7^ MSC treatment. There was a clinical trial that indicated that patients who received 20 million cells were found to have significantly greater LVEF and showed a reduction in scar size in comparison to those who received 200 million MSCs [40]. So, these evidences supported that local treatment with higher dosages of MSCs seems to not result in a higher healing rate. Higher cell concentrations could result in a lower survival rate and/or cell function, and secondly, a larger number of cells could behave immunogenically resulting in increased clearance or deactivation of the cells.

The risk of infection and developing a tumor is the main concern with the use of MSCs. Some studies indicated that MSCs may show a protumorigenic impact on cancers, by inducing neoplastic cell proliferation and promoting angiogenesis [41, 42]. So far, there are no reports of tumors that developed after MSC treatment. However, further clinical trials with long-term follow-up are required to confirm this safety aspect.

There are still many unsolved questions that are concerning. Our study has some limitations: (1) differences in the dose of MSCs injected and the number of injections were not taken into account in the meta-analysis; (2) the time point of fistula healing fluctuates significantly; (3) the best tissue to obtain the MSCs and the amount of these cells being administered in therapy, which may lead to different influences on the growth of MSCs; (4) unclear treatment mechanisms; (5) the most appropriate technique for the MSC transplant is still controversial in the literature (e.g., direct injection, injection with fibrin glue, and delivery on a fistula plug); (6) different criteria for defining fistula healing were applied in the studies; and (7) eligible patients were all at least 18 years of age. The characteristics of patients (such as the age, the gender, and the phase of the disease) are important and those may influence the final effects of MSCs. Due to the limitations of studies, it is difficult to provide recommendations. In the future, we should adopt the optimized treatment scheme on the premise of standardization to enable patients to achieve long-term healing.

## 5. Conclusions

Our study supports that local MSC therapy is emerging as an alternative treatment for complex perianal fistulas. MSC alone or combined with fibrin glue treatment for complex perianal fistula is effective and without risk of anal incontinence or MSC-related AEs. MSCs combined with fibrin glue therapy have a synergistic effect on the healing of fistulas. Local MSC therapy has shown more promising results in fistulas associated with CD than non-CD. Different sources and cell type of MSCs can improve the healing of fistula. MRI to evaluate the healing of fistulas is more objective and accurate. Local treatment with higher dosages of MSCs seems to not result in higher healing rate of fistula. In the future, more RCTs are needed to support our conclusions.

## Figures and Tables

**(a):** 

Study	Type of perianal fistula	*N*	Cell type and source	Outcome	Results (healed)
Garcia-Olmo et al. [14] (2009) Spain	(1) Crohn (2) Cryptoglandular	49	Autologous ASCs	Reepithelialization	17/24 vs. 4/25 CD: 5/7 MSCs+fibrin glue vs. 1/7 fibrin glue at 8 w; cryptoglandular: 12/17 MSCs+fibrin glue vs. 3/18 fibrin glue at 8 w;
Guadalajara et al. [16] (2012) Spain	(1) Crohn (2) Non-CD	30	Autologous ASCs	Reepithelialization+MRI	10/18 vs. 3/12 CD: 2/4 MSCs+fibrin glue vs. 1/2 fibrin glue at 40 m;
Herreros et al. [17] (2012) Spain	Cryptoglandular	183	Autologous ASCs	Reepithelialization+MRI	51/124 vs. 22/59 25/64 MSCs+26/60 MSCs+fibrin glue vs. 22/59 fibrin glue at 24 w
Molendijk et al. [22] (2015) Netherlands	Crohn	21	Allogeneic BMSCs	Reepithelialization+MRI	7/15 MSCs vs. 2/6 saline solution at 12 w
Panés J [23] (2016) Israel	Crohn	212	Allogeneic ASCs	Reepithelialization+MRI	53/103 MSCs vs. 36/101 saline solution at 24 w
Panés J [26] (2018) Israel	Crohn	212	Allogeneic ASCs	Reepithelialization+MRI	58/103 MSCs vs. 39/101 saline solution in 52 w
Garcia-Arranz M [27] (2019) Spain	Cryptoglandular	35	Autologous ASCs	Reepithelialization	10/20 MSCs+fibrin glue vs. 5/19 fibrin glue at 2 years


**(b):** 

Intervention (mean)	Recurrence	Refractory	AEs	Use of concurrent (anti-TNF)
Fixed cell dose	7 months without recurrence	Yes		Yes
First: 2 × 10^7^ MSCs				
Second: 4 × 10^7^ MSCs				
Fixed cell dose	Two completely healed patients were followed up for 4 years without recurrence	Yes		Yes
First: 2 × 10^7^ MSCs				
Second: 4 × 10^7^ MSCs				
Fixed cell dose	Unknown	Yes		No
First: 2 × 10^7^ MSCs				
Second: 4 × 10^7^ MSCs				
Fixed cell dose	24 w without recurrence	Yes		Yes
A: 1 × 10^7^ MSCs				
B: 3 × 10^7^ MSCs				
C: 9 × 10^7^ MSCs				
Fixed cell dose	Being follow-up	Yes	68/103 in MSCs vs. 66/102 in placebo at 24 w	Yes
12 × 10^7^ MSCs				
Fixed cell dose	Being follow-up	Yes	79/103 in MSCs vs. 74/102 in placebo at 52 w	Yes
12 × 10^7^ MSCs				
Fixed cell dose	Recurrence of the fistula was observed at 2-year follow-up in eight previously cured patients	Unknown	7/23 MSCs+fibrin glue vs. 9/21 fibrin glue	No
First: 10 × 10^7^ MSCs				
Second: 10 × 10^7^ MSCs				

## Data Availability

The data used to support the findings of this study are available from the corresponding author upon request.
